# Estrogen Suppresses Osteoclast Function and Cytoskeletal Remodeling via Modulation of the RhoA-Pkn3-c-Src Signaling Pathway in Ovariectomized Rats

**DOI:** 10.3390/biology15181585

**Published:** 2026-09-09

**Authors:** Yuhe Wang, Shipei Yang, Yulu Yuan, Danping Fan, Hongyan Zhao, Meijie Liu

**Affiliations:** Experimental Research Center, China Academy of Chinese Medical Sciences, Beijing 100700, China; 18801094991@163.com (Y.W.); yangsp09@163.com (S.Y.); yuanyulu99@163.com (Y.Y.); fdp0406@gmail.com (D.F.); zhaohongyan1997@163.com (H.Z.)

**Keywords:** postmenopausal osteoporosis, osteoclast, cytoskeleton, estrogen, RhoA-Pkn3-c-Src signaling pathway

## Abstract

Postmenopausal osteoporosis (PMOP) is characterized by excessive bone resorption caused by increased osteoclast activity. Estrogen remains an effective therapeutic option for preventing bone loss; however, its regulatory effects on osteoclast cytoskeletal remodeling have not been fully clarified. In this study, an ovariectomized (OVX) rat model and RANKL-induced RAW264.7 osteoclasts were used to investigate the role of the RhoA-Pkn3-c-Src signaling pathway in the anti-resorptive effects of estrogen. Bone microarchitecture was evaluated by micro-computed tomography, while osteoclast activity and pathway-related proteins were assessed using histological staining and immunofluorescence. In vitro, osteoclast differentiation, bone resorption, actin ring formation, and protein interactions were analyzed, with additional validation using a RhoA agonist. Estrogen treatment markedly alleviated OVX-induced bone loss, reduced osteoclast number and activity, and improved trabecular bone microarchitecture. These changes were accompanied by decreased expression of RhoA, Pkn3, c-Src, and Pyk2 in osteoclasts. Consistently, estrogen suppressed RANKL-induced osteoclast differentiation, bone resorption, and cytoskeletal remodeling in vitro, whereas activation of RhoA partially attenuated these effects. Collectively, these findings suggest that the protective effects of estrogen against osteoclast-mediated bone resorption are associated with modulation of the RhoA-Pkn3-c-Src signaling pathway, providing additional insight into the mechanisms underlying PMOP.

## Simple Summary

Postmenopausal osteoporosis is a common skeletal disorder caused mainly by increased bone loss after estrogen decline. Osteoclasts, the cells responsible for bone resorption, become overactive during estrogen deficiency and contribute to osteoporosis progression. Although estrogen is widely used to prevent bone loss, the mechanisms by which it regulates osteoclast function remain incompletely understood. In this study, we found that estrogen reduced osteoclast activity and protected against bone loss in an ovariectomized rat model. Further experiments showed that estrogen inhibited osteoclast formation, bone resorption, and cytoskeletal remodeling by suppressing the RhoA-Pkn3-c-Src signaling pathway. Activation of RhoA provided additional evidence supporting the involvement of this pathway in estrogen-mediated regulation of osteoclast function. These findings provide new insights into how estrogen regulates osteoclast activity and may contribute to the development of improved strategies for preventing estrogen deficiency-related bone loss.

## 1. Introduction

Postmenopausal osteoporosis (PMOP) is one of the most common metabolic bone disorders affecting aging women and has become a major public health challenge worldwide. It is characterized by progressive loss of bone mass, disruption of bone microarchitecture, and increased susceptibility to fragility fractures, which are primarily driven by estrogen deficiency after menopause [1,2]. In clinical practice, PMOP is mainly diagnosed by assessment of bone mineral density (BMD) using dual-energy X-ray absorptiometry (DXA), combined with evaluation of clinical risk factors and fracture history. Current therapeutic strategies include lifestyle modification, calcium and vitamin D supplementation, and pharmacological interventions such as bisphosphonates, denosumab, anabolic agents, and menopausal hormone therapy. Among these treatments, estrogen replacement therapy has been shown to effectively suppress excessive osteoclast activity, preserve bone mass, and reduce fracture risk by restoring estrogen signaling [3]. However, the application of estrogen therapy remains limited due to potential adverse effects, including increased risks of venous thromboembolism, stroke, and hormone-dependent malignancies in certain populations. Therefore, a better understanding of the mechanisms underlying estrogen-mediated regulation of osteoclast function is essential for developing therapeutic approaches that maintain skeletal benefits while minimizing potential risks.

Osteoclasts (OC) are the principal bone-resorbing cells and play a central role in the development of PMOP [4]. Under physiological conditions, osteoclasts cooperate with osteoblasts to maintain skeletal homeostasis. Efficient bone resorption depends on extensive cytoskeletal remodeling, which enables osteoclasts to establish a polarized morphology and specialized membrane domains, including the ruffled border and sealing zone [5]. These structures create a confined microenvironment for degradation of the mineralized bone matrix. During the resorption cycle, osteoclasts continuously reorganize their actin cytoskeleton, disassembling and reforming actin rings to support cell migration and attachment to new resorption sites. Thus, disruption of cytoskeletal organization impairs the formation of the ruffled border and sealing zone, ultimately leading to reduced bone-resorbing activity [6].

RhoA, a member of the Rho family of small GTPases, is widely recognized as a key regulator of cytoskeletal dynamics in osteoclasts, particularly in podosome organization and actin ring formation required for bone resorption [7,8]. Pkn3 has been suggested to function downstream of RhoA signaling in osteoclasts. Genetic evidence indicates that loss of Pkn3 leads to increased bone mass and attenuated osteoclast-mediated bone resorption, together with defects in F-actin ring formation [9]. At the mechanistic level, activated Pkn3 is reported to accumulate at the sealing zone and facilitate the association between c-Src and proline-rich tyrosine kinase 2 (Pyk2), both of which play essential roles in cytoskeletal organization and the resorptive function of osteoclasts [9]. However, the involvement of the RhoA-Pkn3-c-Src signaling pathway in osteoclast cytoskeletal remodeling under systemic estrogen-deficient conditions has not been fully characterized.

In this study, we used ovariectomized (OVX) rats and RANKL-induced osteoclasts as in vivo and in vitro models. We investigated how estrogen deficiency regulates osteoclast cytoskeletal organization and whether the RhoA-Pkn3-c-Src/Pyk2 signaling pathway contributes to osteoclast functional activation under osteoporotic conditions.

## 2. Materials and Methods

### 2.1. Ovariectomy-Induced Osteoporosis Rat Model

Animal studies were conducted in compliance with the experimental animal ethical guidelines (Ethical No. ERCCACMS21-2309-01). Female Sprague-Dawley (SD) rats (229 ± 11 g, 3 months old) were obtained from Beijing Weitong Lihua Laboratory Animal Technology Co., Ltd., Beijing, China. Experimental animals were reared under standard laboratory conditions (21 ± 2 °C; 50 ± 10% humidity; 12 h light/dark cycle). After a seven-day habituation period, rats were divided into Control (*n* = 12), Sham (*n* = 12), and model (*n* = 24). Ovariectomy was performed on the model group by first administering anesthesia and performing aseptic preparation of the bilateral dorsolateral areas. A 1-cm incision was executed inferior to the rib cage to allow for the ligation of adnexal structures and subsequent bilateral oophorectomy. The surgical site was then sutured layer by layer. In the Sham group, only periovarian adipose tissue was excised, while the remaining surgical procedures were identical to those in the model group. Two weeks post-ovariectomy, the model group was further divided into the ovariectomy group (OVX, *n* = 11; one rat died postoperatively) and the ovariectomy plus estrogen group (OVX+E, *n* = 12). The administration of estradiol valerate (0.0184 mg/mL; Bayer, Leverkusen, Germany) to the OVX+E group was performed through intragastric gavage at a dose of 1 mL/100 g body weight [10,11,12]. Meanwhile, purified water was provided in equivalent volumes to the remaining three groups. The therapeutic regimen was executed once daily for 12 weeks, adhering to a schedule of six treatment days and one day of rest per week. Upon completion of the experimental period, the rats were sacrificed, followed by blood collection via the abdominal aorta and the subsequent harvesting of bilateral femoral and tibial tissues. The collected blood samples were allowed to clot at room temperature and then centrifuged to obtain serum. The serum was heat-inactivated at 56 °C for 30 min, sterilized by filtration through a 0.22-μm membrane, aliquoted, and stored at −80 °C. The prepared serum was subsequently used for the in vitro treatment of RAW264.7 cells.

### 2.2. Micro-Computed Tomography (Micro-CT)

Tibiae from each group were scanned using the Skyscan1276 micro-CT system (Bruker, Billerica, MA, USA). Scanning parameters were: voltage 70 kV, current 200 μA, 0.5 mm Al filter, 10.2 μm resolution, 850 ms exposure time, and a rotation step of 0.6°. The region of interest (ROI), approximately 3 mm in thickness, was defined by selecting 294 consecutive sections distal to the lowest point of the tibial growth plate adjacent to the knee joint. Following the generation of three-dimensional reconstructions via N-Recon software (version 1.6.9.4, Bruker, Billerica, MA, USA), the resulting datasets were imported into the CT-AN platform for the quantification of microarchitectural parameters.

### 2.3. Undecalcified Bone Sections

Rat tibiae were fixed in 4% paraformaldehyde for 3 days, rinsed with running water, and processed through graded ethanol (80%, 90%, absolute ethanol; 3 days each) and xylene (3 days) for dehydration. Bone specimens were infiltrated and embedded in plastic resin, followed by polymerization under vacuum at −20 °C for 10 days. Longitudinal undecalcified sections (5 μm) were cut using a Reichelt Jung 2040 microtome with a tungsten steel knife. Sections were then baked at 60 °C for 4 h and stained with toluidine blue. Parameters measured included trabecular bone volume percentage (TBV%), trabecular resorption surface percentage (TRS%) and trabecular bone formation surface percentage (TFS%).

### 2.4. Immunohistochemistry

Tibiae were fixed in 10% EDTA·Na_2_ solution (pH 7.3) at 25 °C for 3 weeks. Following decalcification, longitudinal sections (6 μm) were cut using a cryostat (Thermo, Waltham, MA, USA), fixed with acetone at 4 °C for 15 min, and dried. Non-specific enzymatic signals were eliminated by exposing the frozen samples to 3% hydrogen peroxide for 15 min to inhibit endogenous peroxidase. Primary antibodies against TRAP (1:200, PA5-116970) and NFATc1 (1:200, PA5-90432) were applied and incubated at 37 °C for 1 h. PBS was used as the negative control in place of the primary antibody. Visualization of the target antigens was achieved through DAB chromogen application following a 30 min treatment with a 1:200 secondary antibody at room temperature. To prevent over-staining, tap water was utilized to quench the reaction immediately. Sections were dehydrated through graded ethanol, cleared with a clearing agent, and mounted with neutral resin. Positive immunoreactivity appeared as brown-yellow particles. The Leica image analysis system was used to randomly select five fields at 400× magnification within the bone marrow cavity to calculate the mean integrated optical density for group comparison.

### 2.5. Measurement of Serum TRAP5b and CTX-I

Circulating levels of TRAP5b (EK12409, SAB, College Park, MD, USA) and CTX-I (EK11957, SAB, College Park, MD, USA) were assessed using commercial ELISA kits after clearing thawed serum of debris through centrifugation (1000 rpm, 3 min).

### 2.6. Transmission Electron Microscopy (TEM)

Bone tissue samples (1–2 mm^3^) from the epiphyseal plate of the femur were fixed with 2.5% glutaraldehyde at 4 °C for 24 h. Following alcohol gradient dehydration, permeabilisation and embedding, ultrathin sections were prepared for observation of the ultrastructure of osteoclast actin rings and the boundaries of the folds under a transmission electron microscope.

### 2.7. Immunofluorescence Double Staining

After returning to room temperature, frozen sections were blocked with 3% hydrogen peroxide for 15 min, followed by permeabilisation and blocking. Sections were incubated overnight at 4 °C with primary antibodies against TRAP (1:100, Ab212723, Abcam, Cambridge, UK) in combination with RhoA (1:100, 38121, SAB, College Park, MD, USA), Pkn3 (1:200, Ab155076, Abcam, Cambridge, UK), c-Src (1:100, 40790, SAB, College Park, MD, USA), or Pyk2 (1:100, 54132, SAB, College Park, MD, USA). PBS was used as a negative control in place of the primary antibody. After PBS washes, the appropriate fluorescent secondary antibody mixture was applied for 1 h in the dark. DAPI was used for nuclear staining, and the sections were examined under a fluorescence microscope. Multi-channel images were captured, where positive signals appeared yellow due to fluorescence overlap, and DAPI-stained nuclei appeared blue. Colocalization analysis was subsequently performed.

### 2.8. Cell Culture and Osteoclast Differentiation Assay

RAW264.7 cells (Chinese Academy of Medical Sciences, Beijing, China) were cultured in DMEM supplemented with 10% fetal bovine serum (FBS). For osteoclast differentiation, RAW264.7 cells were seeded into 96-well plates at a density of 2000 cells/well and cultured in medium containing 50 ng/mL RANKL and 5% serum collected from estrogen-treated OVX rats or vehicle-treated OVX rats. The serum treatment was maintained throughout the entire differentiation period, and the corresponding serum was replenished during medium changes every two days. When osteoclasts appeared, TRAP staining was performed according to the kit instructions (387A-1, Sigma, St. Louis, MO, USA). TRAP staining images were analyzed using ImageJ software (version 1.53e, National Institutes of Health, Bethesda, MD, USA) to quantify osteoclast formation in each group. TRAP activity was measured using a TRAP assay kit. Narciclasine and MPP were dissolved in DMSO to prepare the stock solution, and cells in the OVX group were treated with an equivalent volume of DMSO.

### 2.9. Toluidine Blue Staining

Following established protocols, RAW264.7 cells were co-cultured with bone slices and induced to differentiate for 10 days [13,14]. Bone slices were then removed and immersed in 0.25 mol/L NH_4_OH, ultrasonically cleaned at 42 kHz for 5 min, washed three times with PBS, and blotted dry with filter paper. After a 10-min immersion in toluidine blue, the bone slices were cleansed thrice using distilled water to remove excess dye. The resorption lacuna area was quantified using an image analysis system (Leica DM6000B, Heidelberg, Germany).

### 2.10. Actin Ring Detection

Upon termination of the differentiation protocol, cellular fixation was performed with 4% paraformaldehyde (20 min), followed by a 3-min permeabilization step in 0.1% Triton X-100. F-actin labeling was achieved through a 1-h dark incubation with rhodamine-phalloidin (1:1000, HY-K0903), with DAPI utilized for nuclear identification. Fluorescence images were acquired using a fluorescence microscope. Osteoclasts exhibiting continuous or nearly continuous F-actin rings were considered positive for actin ring formation. For actin ring thickness analysis, six randomly selected osteoclasts were analyzed per group in each independent experiment. The thickness of the F-actin ring was measured using the line measurement tool in ImageJ software, and the mean value was used for subsequent statistical analysis.

### 2.11. Real-Time Quantitative PCR

To assess relative gene expression, total RNA was extracted from induced cells (after 5 days of differentiation and PBS washing) and reverse-transcribed into cDNA. Quantitative PCR was performed using Yeasen-sourced reagents (11141ES60, 11184ES03) under the following cycling conditions: initial denaturation at 95 °C for 2 min, followed by 40 cycles of 95 °C for 10 s and annealing/extension at 60 °C for 30 s. After confirming single-product amplification by melting curve analysis, transcript levels were calculated using the 2^−ΔΔCt^ method. The specific primer sequences are listed in Table 1.

### 2.12. Western Blot Analysis

Cells were lysed in RIPA buffer supplemented with protease and phosphatase inhibitors. Protein concentration was measured using a BCA assay. Equal amounts of protein (20 μg) were separated by SDS-PAGE and transferred to PVDF membranes. The membranes were blocked with 5% BSA and incubated overnight at 4 °C with the indicated primary antibodies, followed by HRP-conjugated secondary antibodies for 1 h at room temperature. Protein signals were detected by ECL, and band density was measured with ImageJ. β-actin served as the loading control.

### 2.13. Co-Immunoprecipitation (Co-IP)

Cell lysates were pre-cleared with normal IgG and Protein A/G magnetic (SC-2003, Santa, Dallas, TX, USA) beads for 1 h at 4 °C to reduce nonspecific binding. After centrifugation, the supernatants were incubated overnight at 4 °C with an anti-c-Src antibody, while normal IgG from the same host species served as the negative control. Protein A/G magnetic beads were then added and incubated for 2 h at 4 °C with gentle rotation. The immune complexes were collected and washed four times with ice-cold lysis buffer. Finally, the bound proteins were eluted with 1× SDS sample buffer containing β-mercaptoethanol by boiling for 10 min and subjected to Western blot analysis.

### 2.14. Statistical Analysis

Statistical analysis was performed using GraphPad Prism software (version 8.0.2, San Diego, CA, USA). Comparisons among multiple groups were performed using one-way analysis of variance (ANOVA), followed by Dunnett’s multiple comparisons test. Differences between groups were considered statistically significant at * *p* < 0.05, ** *p* < 0.01, *** *p* < 0.001 and **** *p* < 0.0001.

## 3. Results

### 3.1. Estrogen Mitigates Bone Density Loss and Improves Bone Microarchitecture in OVX Rats

**Figure 1:**
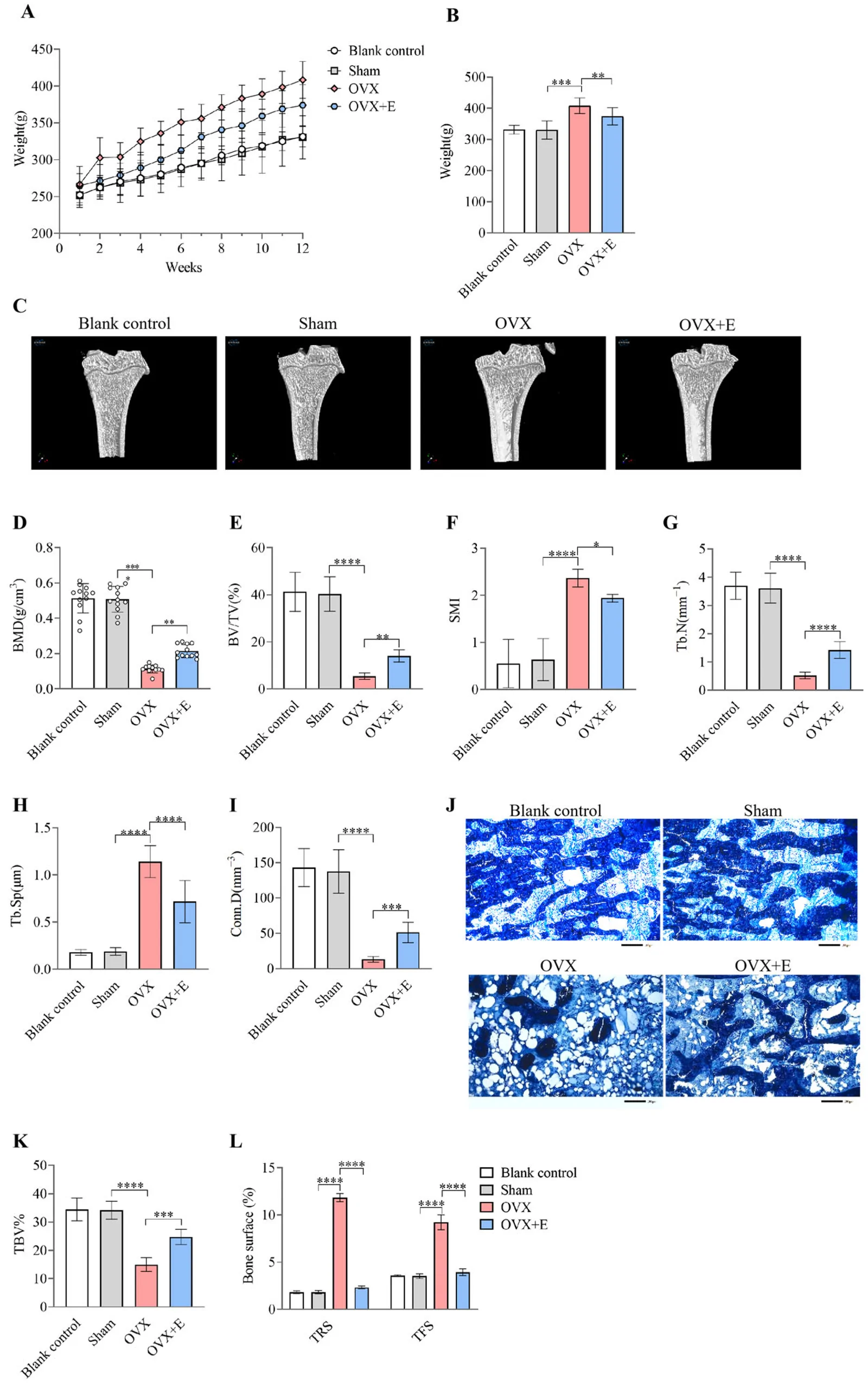
Estrogen mitigates bone density loss and improves bone microarchitecture in OVX rats. (**A**) Changes in body weight during the experimental period. (**B**) Quantification of rat body weight after continuous administration for 12 weeks (*n* = 11–12). (**C**) Micro-CT 3D images of the tibia. (**D**–**I**) Quantitative analysis of BMD, bone volume/tissue volume (BV/TV), structure model index (SMI), trabecular number (Tb.N), trabecular separation (Tb.Sp), and connectivity density (Conn.D) (*n* = 6). (**J**) Representative toluidine blue staining of undecalcified bone sections. Trabecular bone is stained dark blue, whereas osteoid appears light blue. Scale bar = 200 μm. (**K**,**L**) Quantitative analysis of trabecular bone volume/tissue volume (TBV), trabecular bone resorption surface (TRS), and trabecular bone formation surface (TFS) (*n* = 3–4). Data are expressed as mean ± SD. * *p* < 0.05, ** *p* < 0.01, *** *p* < 0.001, **** *p* < 0.0001.

Following the OVX modeling procedure, we measured the body weight and found that the OVX group exhibited a significant increase over time (Figure 1A). After 12 weeks of treatment, the final body weights of the OVX+E group gradually approached those of the sham-operated group (Figure 1B). Micro-CT analysis revealed that bone tissue morphology was more intact following estrogen intervention compared to the OVX group (Figure 1C). Moreover, all bone microstructural parameters in the OVX+E group showed varying degrees of improvement, with increased BMD, BV/TV, Tb.N, and Conn.D, and a reduction in SMI and trabecular spacing (Figure 1D–I). Trabecular bone, the fundamental structural unit of cancellous bone, serves as a key indicator for assessing bone quality. Osteoid, an organic matrix deposited on the bone surface, represents the early stage of bone formation and can reflect the bone-forming function of osteoblasts (OBs). It appears light blue after toluidine blue staining (Figure 1J). Compared with sham-operated controls, the OVX group exhibited a significantly lower BV/TV ratio, underscoring the extensive depletion of trabecular density typical of the osteoporotic marrow environment in this model. Estrogen treatment significantly increased the BV/TV% (Figure 1K). Quantitative analysis found that the percentage of trabecular bone resorption surface (TRS%) was higher than the percentage of trabecular bone formation surface (TFS%), suggesting that the osteoporosis model induced by OVX was a high-turnover osteoporosis model. Estrogen administration reduced these parameters, with a more pronounced inhibitory effect on bone resorption (Figure 1L). These results indicate that estrogen can mitigate bone density loss and improve bone microarchitecture in OVX-induced osteoporosis.

### 3.2. Estrogen Reduces the Formation of OC and Their Ruffled Borders in OVX Rats

**Figure 2:**
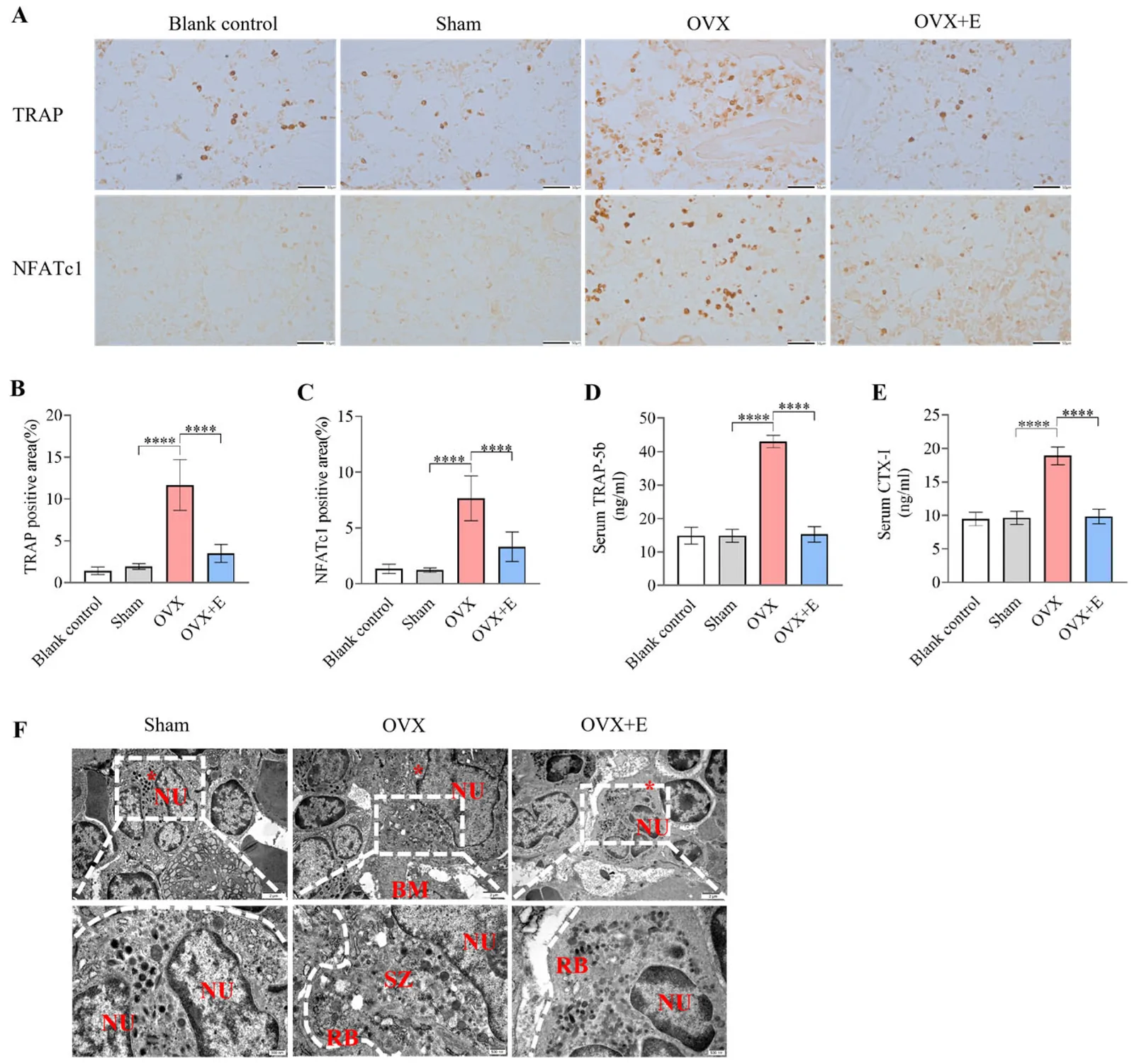
Estrogen reduces osteoclast formation and ruffled border development in OVX rats. (**A**) Representative images of TRAP staining and NFATc1 immunohistochemistry in the tibial bone marrow. Scale bar = 50 μm. (**B**,**C**) Quantitative analysis of TRAP-positive osteoclasts and NFATc1 expression (*n* = 6). (**D**,**E**) Serum levels of TRAP-5b and CTX-I (*n* = 6). (**F**) Representative transmission electron microscopy images of the rat femur. Enlarged views are shown below. *, osteoclast; SZ, sealing zone; BM, bone mineral; RB, ruffled border; NU, nucleus; lines, osteoclast border. Scale bar = 2 μm. Data are expressed as mean ± SD. **** *p* < 0.0001.

To further investigate the effects of estrogen on OC, immunohistochemical staining was used for the osteoclast marker proteins TRAP and NFATc1 (Figure 2A). Statistical analysis revealed that their expression was significantly increased in the tibiae of the OVX group but markedly decreased after estrogen treatment (Figure 2B,C). Consistent with our immunohistochemical findings, ELISA results for the circulating TRAP isoform TRAP5b and CTX-I demonstrated that estrogen significantly reduced the serum levels of both markers compared to the OVX group (Figure 2D,E). During bone resorption, mature OC polarize upon contact with the bone surface and form a sealing zone through the mediation of an actin ring, subsequently developing a ruffled border that adheres to the bone matrix and secretes bone-resorbing substances. Therefore, the formation of the ruffled border reflects the integrity of the actin ring. TEM revealed that OCs in the OVX group exhibited distinct ruffled borders attached to the bone surface. However, estrogen intervention partially reversed the formation of the ruffled border in OCs (Figure 2F). These findings suggest that estrogen has a strong inhibitory effect on osteoclastogenesis and osteoclastic activity.

### 3.3. Estrogen Modulates the RhoA-Pkn3-c-Src Signaling Pathway in OVX Rats

**Figure 3:**
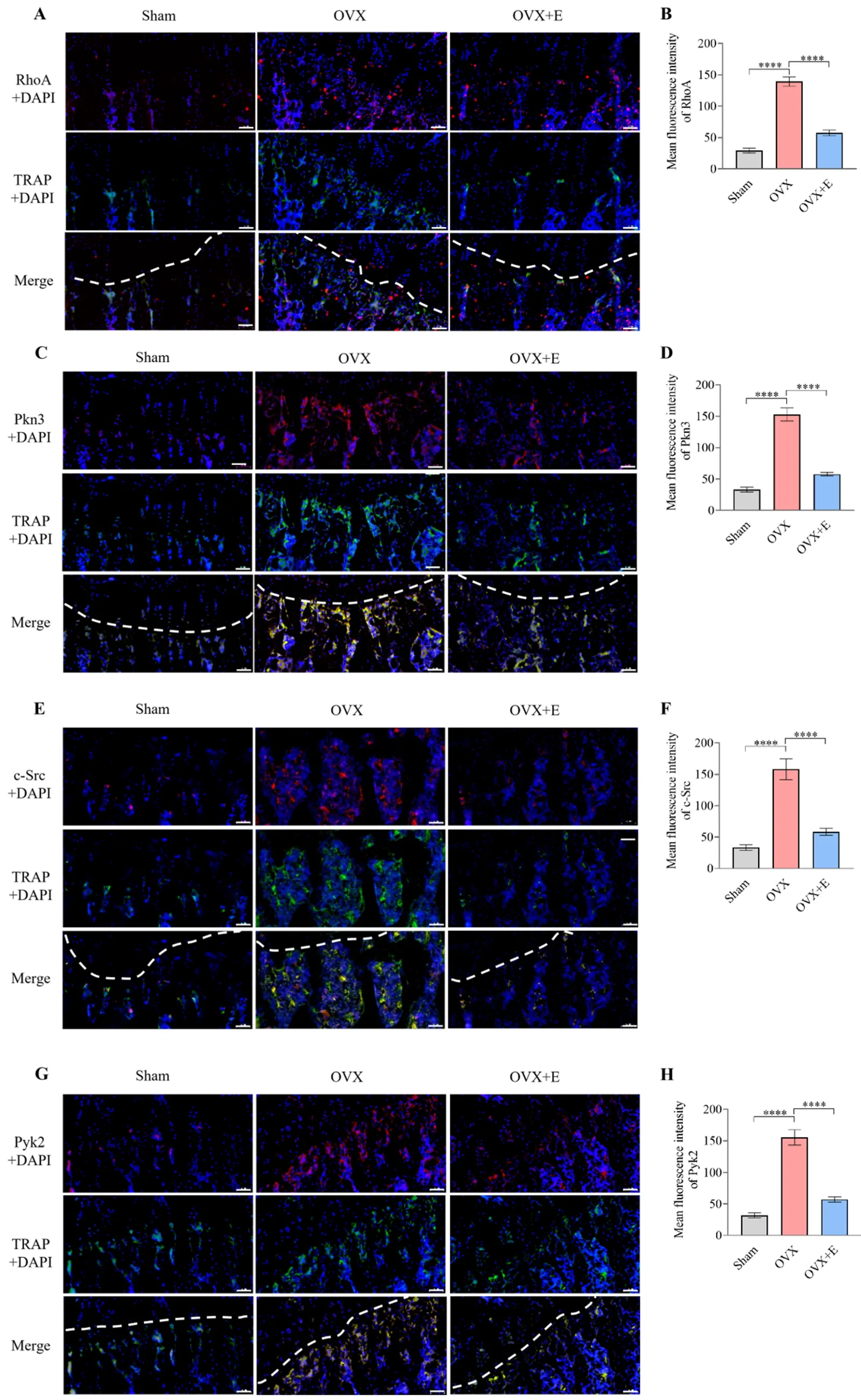
Estrogen modulates the RhoA-Pkn3-c-Src signaling pathway in OVX rats. (**A**) Representative double immunofluorescence images showing RhoA (red), TRAP-positive osteoclasts (green), and colocalization of RhoA and TRAP (yellow). Scale bar = 50 μm. (**B**) Quantitative analysis of the mean fluorescence intensity of RhoA (*n* = 3). (**C**) Representative double immunofluorescence images showing Pkn3 (red), TRAP-positive osteoclasts (green), and colocalization of Pkn3 and TRAP (yellow). Scale bar = 50 μm. (**D**) Quantitative analysis of the mean fluorescence intensity of Pkn3 (*n* = 3). (**E**) Representative double immunofluorescence images showing c-Src (red),TRAP-positive osteoclasts (green), and colocalization of c-Src and TRAP (yellow). Scale bar = 50 μm. (**F**) Quantitative analysis of the mean fluorescence intensity of c-Src (*n* = 3). (**G**) Representative double immunofluorescence images showing Pyk2 (red), TRAP-positive osteoclasts (green), and colocalization of Pyk2 and TRAP (yellow). Scale bar = 50 μm. (**H**) Quantitative analysis of the mean fluorescence intensity of Pyk2 (*n* = 3). The white lines indicate the tibial growth plate. Data are expressed as mean ± SD. **** *p* < 0.0001.

The RhoA-Pkn3-c-Src signaling pathway plays a profound role in regulating the dynamics of OC cytoskeletal structures, particularly the actin ring. To further examine the molecular mechanism by which estrogen influences osteoclasts, double immunofluorescence staining was performed. TRAP protein was labeled with green fluorescence, while target proteins were labeled with red fluorescence (Figure 3A,C,E,G). The extent of protein expression was quantified by measuring the mean fluorescence intensity of RhoA, Pkn3, c-Src, and Pyk2 specifically within TRAP-positive osteoclasts (Figure 3B,D,F,H). The results showed that the protein expression levels of RhoA, Pkn3, c-Src, and Pyk2 in OCs from the OVX group were significantly higher than those in the Sham group. Following estrogen intervention, the expression of these proteins markedly decreased. These findings indicate that estrogen can attenuate the enhanced osteoclast activity induced by ovariectomy by modulating the RhoA-Pkn3-c-Src signaling pathway.

### 3.4. Estrogen-Mediated Attenuation of Osteoclastic Function and F-Actin Cytoskeletal Assembly In Vitro

**Figure 4:**
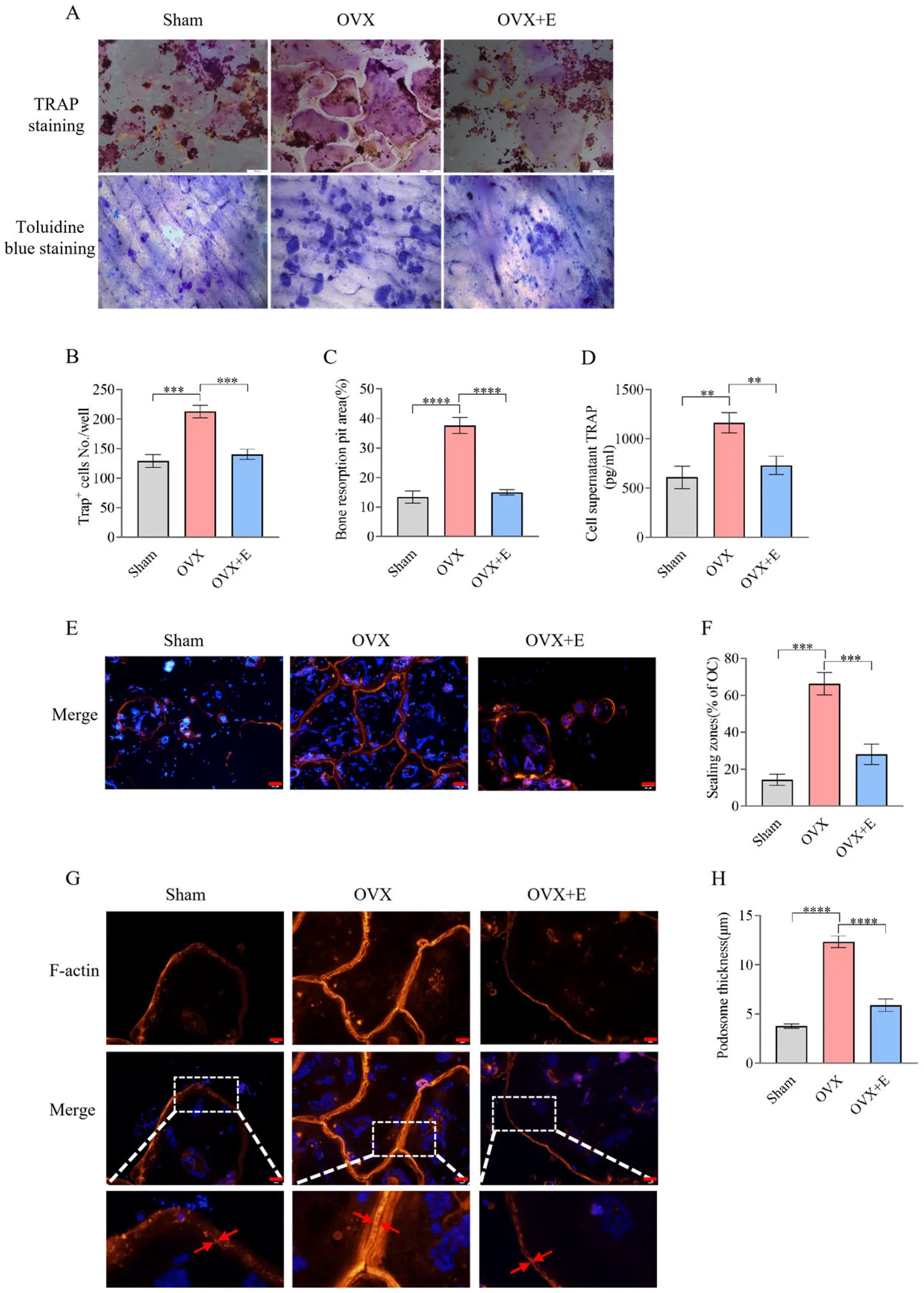
Estrogen suppresses osteoclast activity and F-actin ring formation in vitro. (**A**) Representative tartrate-resistant acid phosphatase (TRAP) staining images of RANKL-induced osteoclasts and toluidine blue staining of bone slices after 10 days of differentiation. Pink indicates TRAP-positive cells, and blue-purple indicates bone resorption pits. Scale bar = 200 μm. (**B**) Quantitative analysis of TRAP-positive osteoclasts (*n* = 3). (**C**) Quantitative analysis of bone resorption pit area (*n* = 3). (**D**) TRAP levels in osteoclast culture supernatants measured by ELISA (*n* = 3). (**E**) Representative images of F-actin staining with rhodamine-phalloidin and DAPI counterstaining in RANKL-induced osteoclasts. Scale bar = 100 μm. (**F**) The percentage of osteoclasts with F-actin ring sealing zones among the total number of osteoclasts. Orange indicates F-actin rings. (*n* = 3). (**G**) Representative images of osteoclast actin rings stained with rhodamine-phalloidin. Enlarged views are shown below. Red arrows indicate the thickness of the F-actin rings. Scale bar = 50 μm. (**H**) Quantitative analysis of actin ring thickness (*n* = 3). Data are expressed as mean ± SD. ** *p* < 0.01, *** *p* < 0.001, **** *p* < 0.0001.

TRAP staining revealed a significant reduction in the number of mature osteoclasts in the estrogen-treated group. Bone resorption assays demonstrated that estrogen effectively decreased the formation of resorption pits (Figure 4A–C). Consistent with these findings, ELISA determination of TRAP levels in the cell culture supernatant showed that estrogen markedly reduced the levels of this osteoclast marker (Figure 4D). F-actin ring staining indicated that while RANKL stimulation induced distinct F-actin rings in osteoclasts, treatment with estrogen resulted in a reduction in intact F-actin ring formation (Figure 4E,F). Further investigation revealed that the thickness of F-actin rings was significantly decreased after estrogen intervention, indicating that estrogen impairs F-actin ring assembly (Figure 4G,H).

### 3.5. Estrogen Modulates the RhoA-Pkn3-c-Src Signaling Pathway in RANKL-Induced Osteoclasts

**Figure 5:**
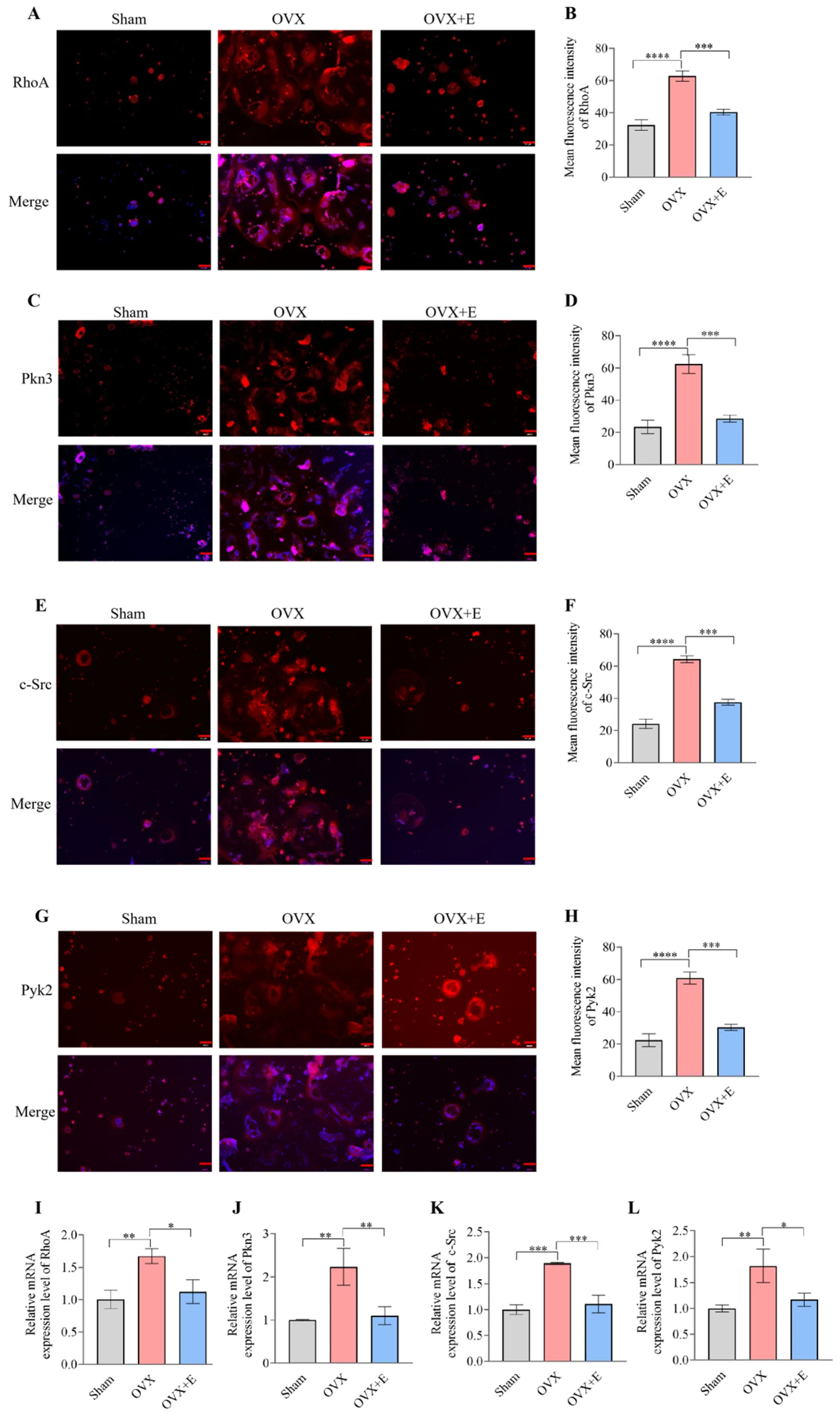
Estrogen reduces the expression of RhoA-Pkn3-c-Src signaling pathway in RANKL-induced osteoclasts. (**A**,**C**,**E**,**G**) Representative immunofluorescence images showing the expression of RhoA, Pkn3, c-Src, and Pyk2 (red) in RANKL-induced osteoclasts. Nuclei were counterstained with DAPI (blue). Scale bar = 100 μm. (**B**,**D**,**F**,**H**) Quantitative analysis of the mean fluorescence intensity of RhoA, Pkn3, c-Src, and Pyk2 (*n* = 3). (**I**–**L**) Relative mRNA expression levels of *RhoA*, *Pkn3*, *c-Src*, and *Pyk2* (*n* = 3). Data are expressed as mean ± SD. * *p* < 0.05, ** *p* < 0.01, *** *p* < 0.001, **** *p* < 0.0001.

To further investigate the mechanism by which estrogen regulates OC cytoskeletal reorganization by influencing actin ring formation, we determined the expression of the RhoA-Pkn3-c-Src signaling pathway in a cellular model. Immunofluorescence staining results showed that the RhoA-Pkn3-c-Src signaling pathway was upregulated in RANKL-induced osteoclasts, while estrogen treatment markedly reduced their expression levels (Figure 5A–H). Further analysis by RT-qPCR yielded consistent findings, demonstrating that estrogen downregulated the expression levels of related genes (Figure 5I–L).

### 3.6. Estrogen Can Modulate Osteoclast Formation Induced by the RhoA Agonist Narciclasine

**Figure 6:**
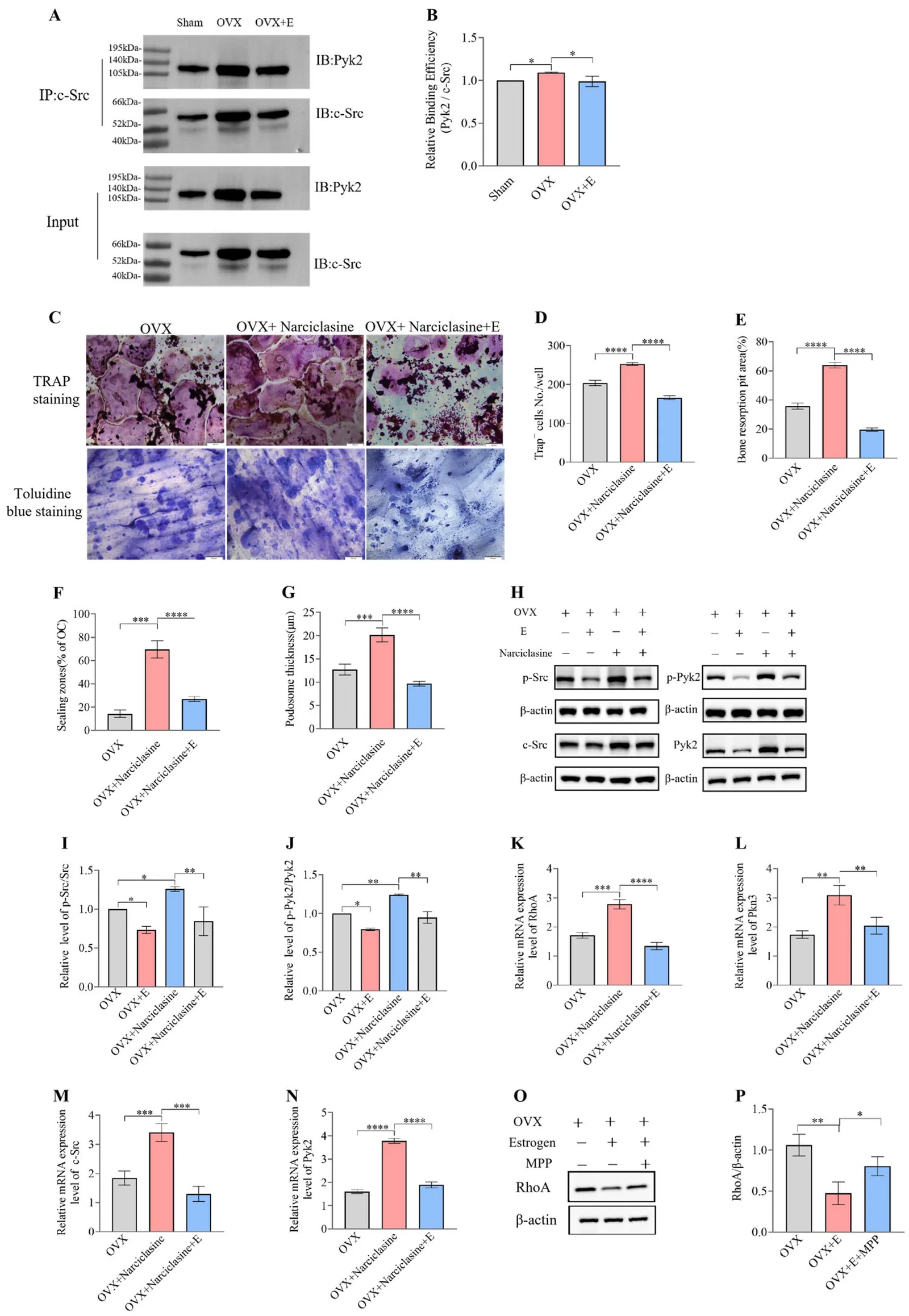
Estrogen attenuates RhoA agonist-induced osteoclast formation and bone resorption. (**A**) Representative co-immunoprecipitation analysis of the interaction between c-Src and Pyk2. (**B**) Quantitative analysis of the relative binding efficiency between c-Src and Pyk2. The relative binding efficiency was calculated using the ratio-of-ratios method: (IP: Pyk2/IP: c-Src)/(Input: Pyk2/Input: c-Src), and then normalized to the Sham group (*n* = 3). (**C**) Representative TRAP staining images of RANKL-induced osteoclasts and toluidine blue staining of bone slices after 10 days of differentiation. Pink indicates TRAP-positive cells, and blue-purple indicates bone resorption pits. Scale bar = 200 μm. (**D**) Quantitative analysis of TRAP-positive osteoclasts. (*n* = 3) (**E**) Quantitative analysis of bone resorption pit area (*n* = 3). (**F**) The percentage of osteoclasts with F-actin ring sealing zones among the total number of osteoclasts (*n* = 3). (**G**) Quantitative analysis of actin ring thickness (*n* = 3). (**H**) Representative Western blot images showing the expression of total Src, phosphorylated Src (p-Src), total Pyk2, and phosphorylated Pyk2 (p-Pyk2) in RANKL-induced osteoclasts following the indicated treatments. (**I**) Relative phosphorylation level of Src, expressed as the ratio of p-Src to total Src (p-Src/Src) (*n* = 3). (**J**) Relative phosphorylation level of Pyk2, expressed as the ratio of p-Pyk2 to total Pyk2 (p-Pyk2/Pyk2) (*n* = 3). (**K**–**N**) Relative mRNA expression levels of *RhoA*, *Pkn3*, *c-Src*, and *Pyk2* (*n* = 3). (**O**) Western blot images showing RhoA expression in osteoclasts cultured with RANKL and MPP (5 µM). (**P**) Quantitative analysis of RhoA/β-actin ratio (*n* = 3). Data are expressed as mean ± SD. * *p* < 0.05, ** *p* < 0.01, *** *p* < 0.001, **** *p* < 0.0001.

Studies have shown that RhoA promotes the formation of a Pkn3 and c-Src complex, which facilitates c-Src activation and enhances sealing zone assembly and the bone-resorbing capacity of osteoclasts. Therefore, we validated this interaction through Co-IP assays. The interaction between c-Src and Pkn3 was enhanced in the OVX group, whereas the amount of Pyk2 co-immunoprecipitated with c-Src was significantly decreased in the estrogen group (Figure 6A,B; Supplementary Figure S1). Given that estrogen modulates the RhoA-Pkn3-c-Src signaling pathway during RANKL-induced osteoclast differentiation, we used the agonist narciclasine. Treatment with 0.1 μM narciclasine significantly promoted the differentiation potential of osteoclasts. Following estrogen intervention, the differentiation and bone resorption capacity of OCs were still reduced. Estrogen reduced the number of mature osteoclasts (Figure 6C–E). Narciclasine facilitated the formation of the osteoclast actin ring, leading to a significant increase in the number of mature osteoclasts and a marked thickening of the actin ring (Figure 6F,G). To further examine changes in the RhoA-Pkn3-c-Src signaling pathway, we performed Western blot analysis to assess phosphorylated protein levels of c-Src and Pyk2. Estrogen treatment markedly reduced the phosphorylated levels (p-Src and p-Pyk2). In contrast, activation of RhoA significantly increased phosphorylated levels of these proteins. After co-treatment with estrogen, the RhoA agonist-induced increase in phosphorylation levels was markedly reversed and was significantly lower than that in the agonist-only group. These results suggest that estrogen suppresses RhoA-mediated activation of the RhoA-Pkn3-c-Src signaling pathway in osteoclast formation (Figure 6H–J; Supplementary Figure S2). RT-qPCR analysis indicated a decrease in the mRNA expression levels of genes associated with the RhoA-Pkn3-c-Src signaling pathway following estrogen treatment (Figure 6K–N). To further investigate whether ERα mediates the effect of estrogen on RhoA, cells were treated with the selective ERα antagonist MPP. Western blot analysis showed that estrogen treatment significantly reduced RhoA protein levels, whereas co-treatment with MPP markedly attenuated the inhibitory effect of estrogen on RhoA protein expression (Figure 6O,P; Supplementary Figure S3). These findings suggest that ERα is involved, at least in part, in mediating the suppressive effect of estrogen on RhoA protein expression.

## 4. Discussion

PMOP is characterized by accelerated bone turnover resulting from estrogen deficiency, in which excessive osteoclast-mediated bone resorption exceeds compensatory bone formation and ultimately leads to progressive bone loss [15,16]. Consistent with this pathological feature, OVX rats in the present study exhibited marked deterioration of bone microarchitecture, accompanied by increased osteoclast activity and enhanced bone turnover. Estrogen treatment effectively attenuated these changes, as evidenced by improvements in trabecular bone parameters and a reduction in osteoclast-mediated bone resorption. Histological analyses further indicated that estrogen partially restored the imbalance between bone resorption and bone formation observed in OVX animals. These findings support the protective role of estrogen in maintaining skeletal homeostasis and are consistent with previous reports demonstrating the anti-resorptive effects of estrogen in PMOP.

Bone resorption is a highly coordinated process that depends on the dynamic regulation of osteoclast morphology and cytoskeletal organization. Among these events, actin ring formation is essential for establishing the sealing zone required for efficient bone resorption [17,18]. Consistent with previous studies, RANKL stimulation markedly promoted osteoclast differentiation and cytoskeletal remodeling, as reflected by enhanced actin ring formation [19]. In the present study, estrogen deficiency was associated with increased osteoclast activity and more pronounced cytoskeletal organization both in vivo and in vitro. Conversely, estrogen treatment reduced actin ring formation and attenuated osteoclast-mediated bone resorption. These observations suggest that the anti-resorptive effects of estrogen may be partly related to its ability to modulate osteoclast cytoskeletal remodeling.

Cytoskeletal remodeling is required throughout osteoclast differentiation and activation, and disruption of this process directly affects bone-resorptive capacity. The RhoA-Pkn3-c-Src signaling pathway has been implicated in the regulation of osteoclast cytoskeletal organization and sealing zone formation [20]. In the present study, double immunofluorescence analyses demonstrated increased expression of RhoA, Pkn3, c-Src, and Pyk2 in osteoclasts from OVX rats, which was accompanied by enhanced osteoclast activity and bone resorption. Estrogen treatment reduced the expression of these molecules while attenuating osteoclast activity. Similar observations have been reported in previous studies, in which suppression of RhoA signaling impaired sealing zone organization and osteoclast function [18,21]. Together with the in vitro findings, these results suggest that modulation of the RhoA-Pkn3-c-Src signaling axis may contribute to the inhibitory effects of estrogen on osteoclast cytoskeletal remodeling and bone resorption.

However, some limitations should be considered in the present study. Firstly, the in vitro experiments were mainly performed using RAW264.7 cells, which may not fully reflect the physiological responses of primary osteoclast precursors. Future studies using primary BMM-derived osteoclasts will be important to further validate these findings in a more physiologically relevant model. Secondly, although pharmacological modulation and downstream signaling analyses supported the involvement of the RhoA-Pkn3-c-Src pathway, further genetic validation would strengthen the causal relationship between this signaling axis and estrogen-mediated regulation of osteoclast function. Nevertheless, the in vitro and in vivo findings together provide complementary evidence that the RhoA-Pkn3-c-Src signaling pathway plays a critical role in osteoclast cytoskeletal remodeling and bone resorption.

## 5. Conclusions

**Figure 7:**
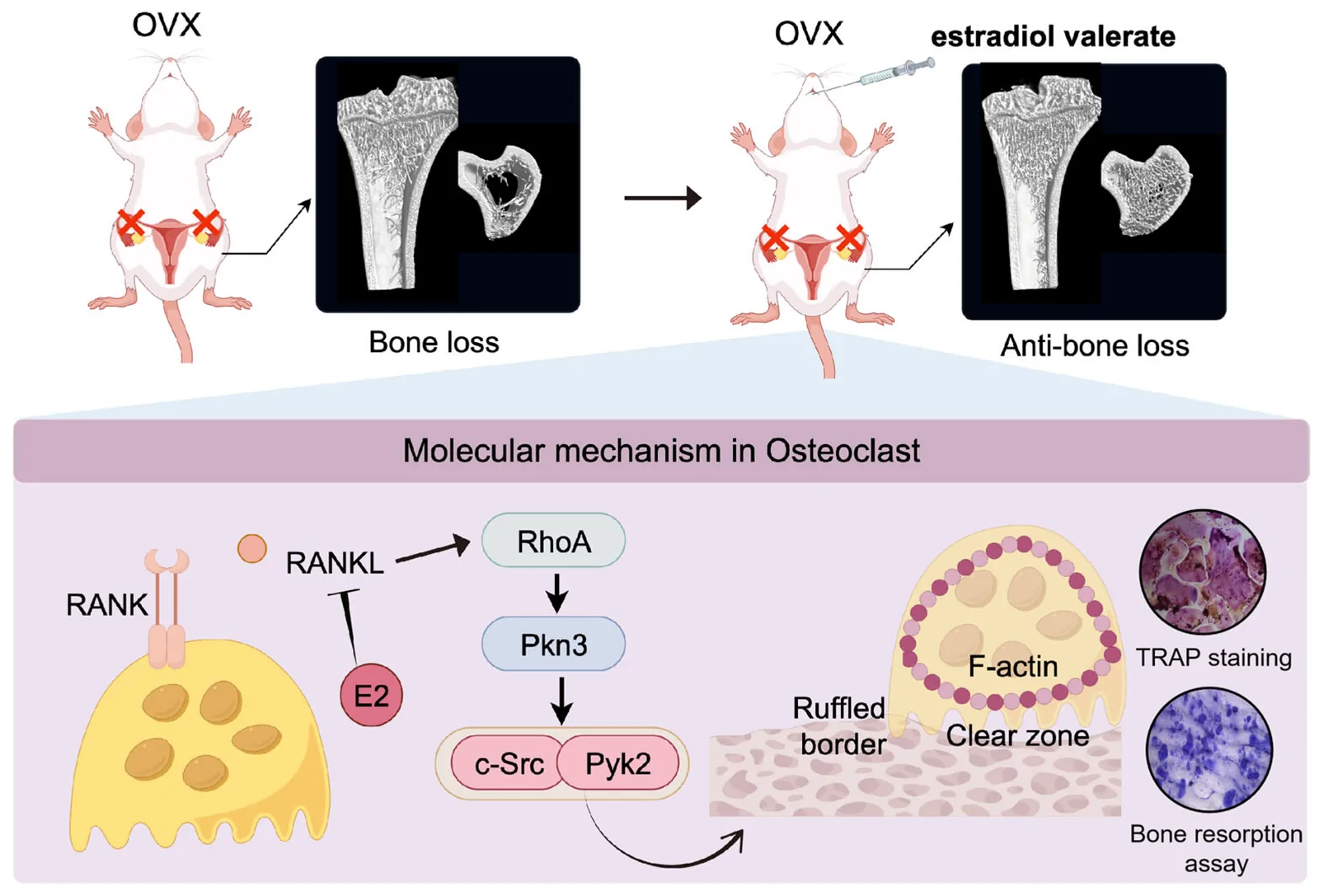
Estrogen suppresses osteoclast cytoskeletal remodeling through the RhoA-PKN3-c-Src signaling pathway.

In summary, estrogen attenuated OVX-induced bone loss and suppressed osteoclast-mediated bone resorption both in vivo and in vitro. These effects were accompanied by reduced osteoclast cytoskeletal remodeling and decreased expression of the RhoA-Pkn3-c-Src signaling pathway. Our findings support a potential role for this pathway in the anti-resorptive actions of estrogen in osteoclasts and suggest that it may be a potential target for the treatment of PMOP (Figure 7). These findings may help to clarify how estrogen regulates osteoclast activity and provide a basis for exploring new therapeutic approaches for PMOP beyond conventional estrogen replacement therapy.

## Figures and Tables

**Table 1:** Primer sequences used in RT-qPCR experiments.

Gene	Primer Sequences: (Forward)	Primer Sequences: (Reverse)	Amplicon Length (bp)
*RhoA*	GGTCTATGTGCCCACGGTGTTTG	GGCGGTCATAATCTTCCTGTCCAG	110
*Pkn3*	CGGATGAAGATAAGCAGCCTGGAG	AGCCACAGCAGCCTCTACTCG	105
*c-Src*	TCACCGCCTCACTACCGTATGTC	CATCCACACCTCTCCGAAGCAAC	136
*Pyk2*	GCACAGTCCATTCAGCCCACAG	TCACCACCACCACATAGTCCTCAG	142
*GAPDH*	GGTTGTCTCCTGCGACTTCA	TGGTCCAGGGTTTCTTACTCC	81

## Data Availability

The original contributions presented in this study are included in the article/Supplementary Materials. Further inquiries can be directed to the corresponding author. Figure 7 in this article was supported by Figdraw (ID: RUWUS11daa).

## Supplementary Materials

The following supporting information can be downloaded at: https://www.mdpi.com/article/10.3390/biology15181585/s1, Figure S1: Original uncropped Western blot images corresponding to Figure 6A; Figure S2: Original uncropped Western blot images corresponding to Figure 6H; Figure S3: Original uncropped Western blot images corresponding to Figure 6O.
