# Supplementary material for: Estrogen Suppresses Osteoclast Function and Cytoskeletal Remodeling via Modulation of the RhoA-Pkn3-c-Src Signaling Pathway in Ovariectomized Rats
Source: Biology (Basel). 2026 Sep 9;15(18):1585. doi: 10.3390/biology15181585 (PMC13603641; doi:10.3390/biology15181585)

Supplementary Figure S1 Original uncropped western blot images corresponding to Figure 6A

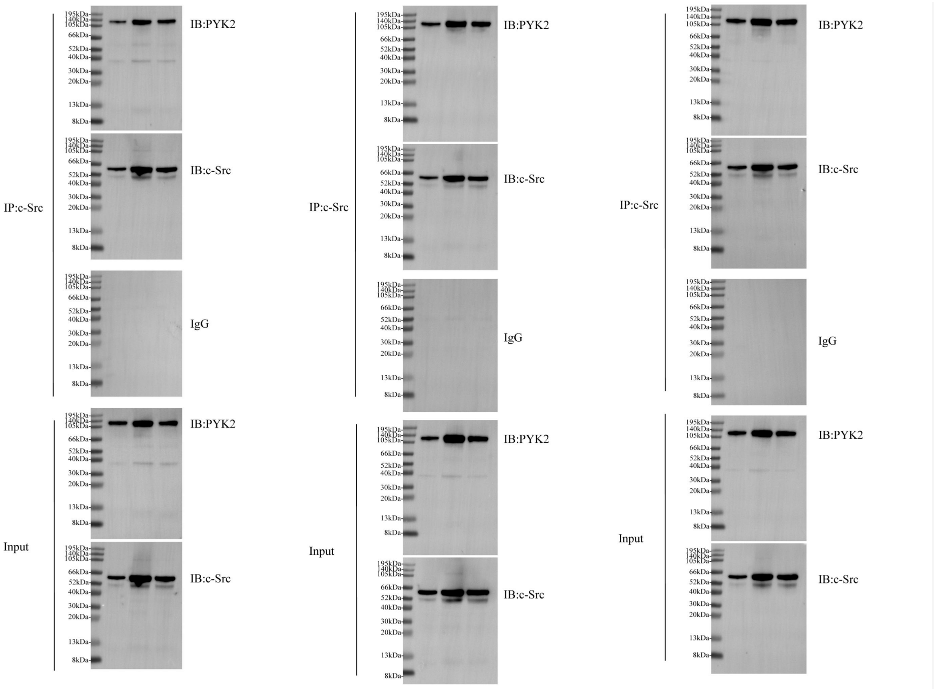

Supplementary Figure S2 Original uncropped western blot images corresponding to Figure 6H

The loading β-actin and target proteins were detected sequentially on the same membrane by stripping and reprobing. Therefore, they were imaged separately but originated from the same membrane.

**c-Src** **β-actin**

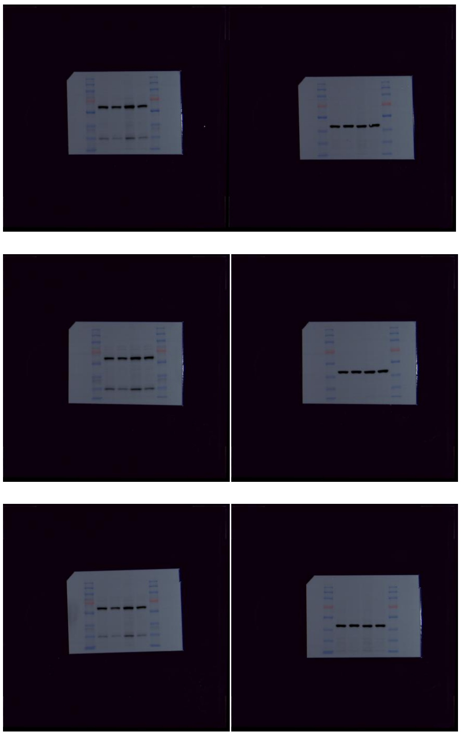

**p-Src** **β-actin**

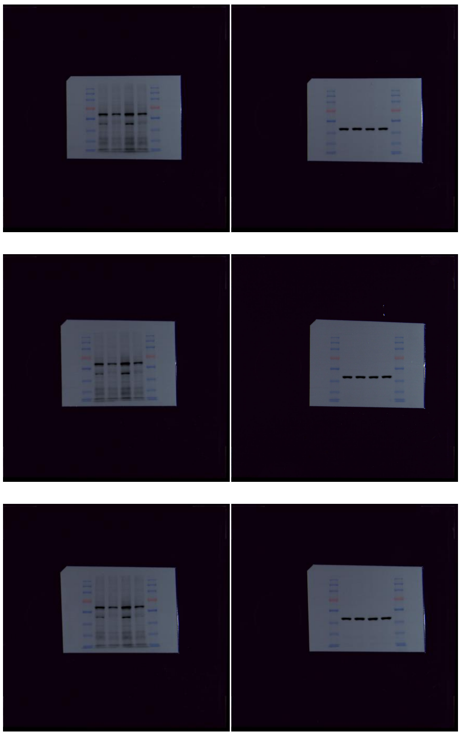

**Pyk2** **β-actin**

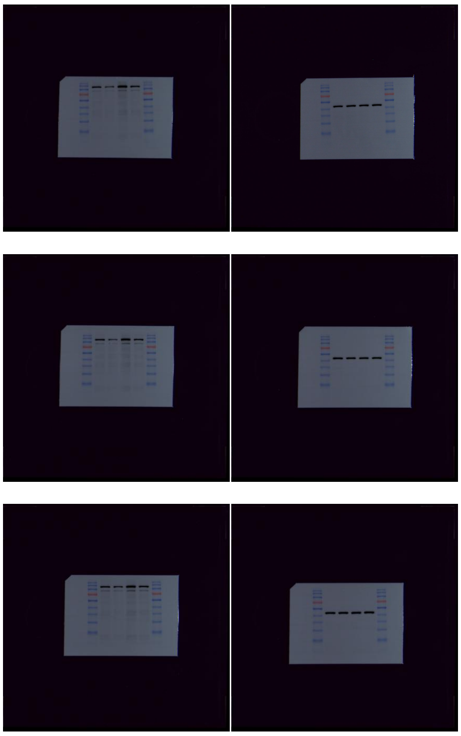

**P-Pyk2** **β-actin**

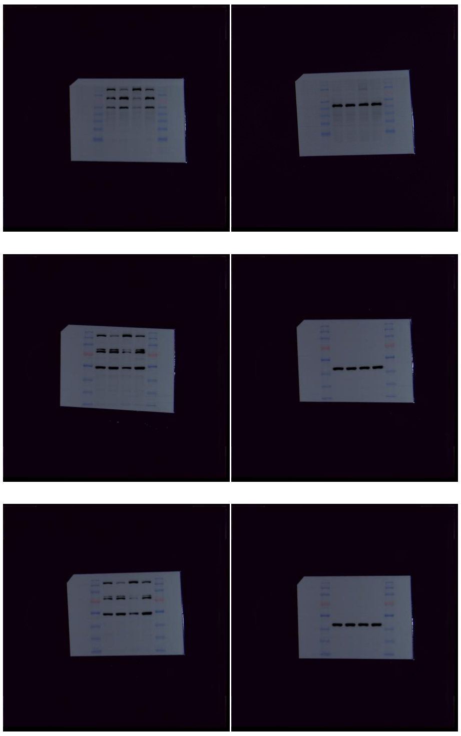

Supplementary Figure S3 Original uncropped western blot images corresponding to Figure 6O

The loading β-actin and target proteins were detected sequentially on the same membrane by stripping and reprobing. Therefore, they were imaged separately but originated from the same membrane.

**RhoA** **β-actin**

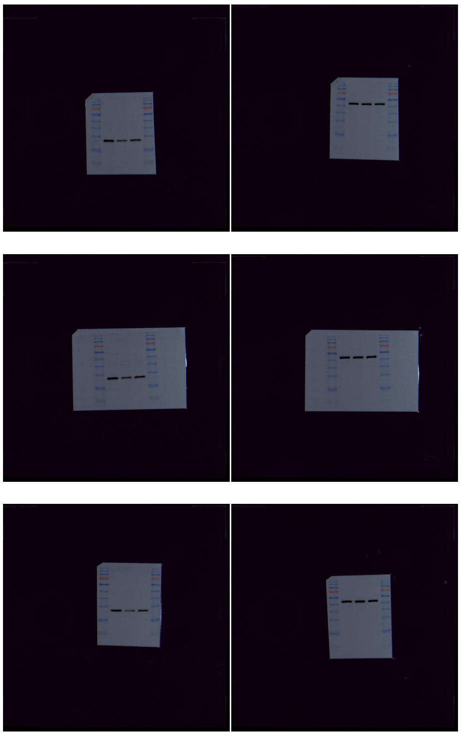

Supplement: Supplementary file 1 [file biology-15-01585-s001.zip › biology-4469767-supplementary.pdf]
